# Effects of local and systemic treatment with human natural killer-1 mimetic peptide (HNK-1) after ventral root avulsion and reimplantation in mice

**DOI:** 10.1590/1678-9199-JVATITD-2023-0065

**Published:** 2024-05-20

**Authors:** Natalia Scanavachia da Silva, Julia Lombardi, Frank Kirchhoff, Rui Seabra Ferreira, Benedito Barraviera, Alexandre Leite Rodrigues de Oliveira, Luciana Politti Cartarozzi

**Affiliations:** 1 Department of Structural and Functional Biology, Laboratory of Nerve Regeneration, Institute of Biology, University of Campinas (Unicamp), Campinas, SP, Brazil; 2 Molecular Physiology, Center for Integrative Physiology and Molecular Medicine (CIPMM), University of Saarland, Homburg, Germany.; 3 Center for Gender-specific Biology and Medicine (CGBM), University of Saarland, Homburg, Germany.; 4 Center for the Study of Venoms and Venomous Animals (CEVAP), São Paulo State University (UNESP), Botucatu, SP, Brazil.

**Keywords:** HNK-1 mimetic peptide, Ursolic acid, Heterologous fibrin biopolymer, Neuroprotection, Immunomodulation

## Abstract

**Background:**

Spinal ventral root injuries generate significant motoneuron degeneration, which hinders full functional recovery. The poor prognosis of functional recovery can be attributed to the use or combination of different therapeutic approaches. Several molecules have been screened as potential treatments in combination with surgical reimplantation of the avulsed roots, the gold standard approach for such injuries. Among the studied molecules, human natural killer-1 (HNK-1) stands out as it is related to the stimulation of motor axon outgrowth. Therefore, we aimed to comparatively investigate the effects of local administration of an HNK-1 mimetic peptide (mp-HNK-1) and systemic treatment with ursolic acid (UA), another HNK-1 mimetic, after ventral root avulsion and reimplantation with heterologous fibrin biopolymer (HFB).

**Methods:**

Female mice of the isogenic strain C57BL/6JUnib were divided into five experimental groups: Avulsion, Reimplantation, mp-HNK-1 (in situ), and UA (systemic treatment). Mice were evaluated 2 and 12 weeks after surgery. Functional assessment was performed every four days using the Catwalk platform. Neuronal survival was analyzed by cytochemistry, and glial reactions and synaptic coverage were evaluated by immunofluorescence.

**Results:**

Treatment with UA elicited long-term neuroprotection, accompanied by a decrease in microglial reactions, and reactive astrogliosis. The neuroprotective effects of UA were preceded by increased glutamatergic and GABAergic inputs in the ventral spinal cord two weeks after injury. However, a single application of mp-HNK-1 had no significant effects. Functional analysis showed that UA treatment led to an improvement in motor and sensory recovery.

**Conclusion:**

Overall, the results indicate that UA is neuroprotective, acting on glial cells and synaptic maintenance, and the combination of these findings led to a better functional recovery.

## Background

Proximal nerve injuries occur often within the spinal canal and therefore close to the cell body, giving rise to significant cell death. Patients who had plexus injury with the root avulsion are regarded as suffering from a longitudinal spinal cord injury and therefore considered not amendable to repair with a functional outcome. Thus, restoration of limb function and control of pain after such injuries are often frustrating tasks [1-5].

The complete avulsion of the ventral root from the spinal cord surface will trigger a cascade of subcellular processes leading to rapid motoneuron death [6]. For that reason, the repair of the avulsed root must be done urgently, as it can interfere with cell death. Such a repairing process is possible because the root itself reacts as a peripheral nerve thus enabling functional recovery if repaired. One strategy to promote regeneration in those cases is to implant the root back into the spinal cord surface. The reimplantation of the avulsed roots has been studied for decades, even reaching application in humans [5, 7-9].

A basic requirement for functional recovery after an avulsion injury is the survival of the motoneurons and further axonal regrowth towards the target muscle and, for that, the regenerating motor axons need to pass towards the root, from the ventral horn through a region of scaring. Such scar is caused by avulsion and reimplantation and is composed of a trabecular structure of astrocytic processes and extracellular matrix (ECM) molecules, somehow different from other scars in the central nervous system, especially for having a unique content of tissue components and substances that are characteristic of the peripheral nerve [1, 2, 4].

Despite the encouraging results that the surgical reimplantation of the avulsed roots has reached over the years, the functional outcome after such a procedure is still poor, especially considering the limb distal musculature [10, 11]. So, other therapeutic approaches that can be combined with reimplantation are brought to light, aiming to establish synergistic neuroprotective and regenerative effects.

For that, the use of animal models is crucial, as they recapitulate the primary and secondary injury processes. Studies of ventral root avulsion and reimplantation were extensively conducted in monkeys, cats, and rats, but there is a lack of such studies in mice [8, 12-16]. The relevance of having a mouse-based model of root lesion and repair lies in the smaller size for handling allowing *in vivo* imaging [17]. This is coupled with the fact that mice are prompt to genetic manipulation and have been extensively used in the development of transgenic and humanized models [18].

The human natural killer-1 (HNK-1) carbohydrate is a sulfated trisaccharide HSO3-3GlcAβ1-3Galβ1-4GlcNAc. The antigen HNK-1 was initially recognized on the surface of human natural killer cells [19], and later studies have indicated that HNK-1 displays controlled expression and distinctive functions within the nervous system, such as neuritogenesis, myelination, synaptic plasticity, post-injury regeneration, learning, and memory [20-27]. Only a limited number of proteins have been suggested as potential receptors, including laminins, cadherin-2, and high-mobility group box (HMGB) 1 and 2. These distinct protein groups are associated with various functions of HNK-1, such as cell migration and neurite outgrowth, which promote neuronal and glial cell adhesion to the ECM. Also, it correlates with synaptic plasticity by biding to cadherin-2, which interacts with the GluA2 subunit of the AMPA-receptor in the presence of HNK-1; and neurite outgrowth by interacting with HMGB [28].

Despite the significance of HNK-1 for motor fiber growth and regeneration, there is no robust information on proximal root lesions, which take place after brachial and lumbosacral plexuses avulsion. Of note, within the peripheral nervous system, HNK-1 is detected in myelinating Schwann cells associated with motor axons but not in cells associated with sensory axons, indicating a potential specific role of HNK-1 in the outgrowth/regrowth of motor axons [29, 30]. Due to its specific association with motor axons, HNK-1 emerges as a promising candidate for promoting motor regeneration following injuries, such as ventral root avulsion.

Due to its biological importance, it is desirable to have sufficient amounts of HNK-1 to elucidate its functional roles further. For that, it is important to synthesize mimetic peptides, as they have a composition whose essential elements mimic a natural peptide or protein, retaining the ability to interact with the target and producing the same biological effect [31-35]. The peptides are composed of short chains with one hundred amino acids or less, lack tertiary structure and stand out for being diversified in functional terms, as they act as hormones or releasing factors, neuropeptides, anti-infective agents, growth factors, and are also known to influence vital physiological processes via inter- and intracellular communication and signal transduction mediated by several classes of transmembrane receptors [36-38].

The mimetic peptide of HNK-1 (mp-HNK-1) has a peptide sequence of 15 amino acids (FHTRLFVSDWYHTP). It has shown promising results for nerve regeneration, stimulating motoneuron neurite outgrowth, leading to greater neuronal survival and regeneration of motor axons in a similar manner as the HNK-1 glycan [29, 34, 39, 40].

Moreover, small organic substances that mimic structurally and functionally the HNK-1 were identified [41, 42]. Among them, the Ursolic Acid (UA) stands out. UA is a triterpenoid compound (C_30_H_48_O_3_) with a molecular weight of 456.68 g/mol, which belongs to the class of isoprenoid compound C30. UA, naturally occurring in plants extensively utilized as medicinal herbs and food sources [43, 44], is relatively non-toxic and commonly used in cosmetics and health products [45, 46]. In addition, UA has been experimentally tested after sciatic nerve injury and traumatic brain injury models, demonstrating promising results, especially due to its antioxidant and immunomodulatory effects [45, 47].

Thus, the association of strategies seeking to favor neuronal survival and stimulate motor axonal regeneration is of fundamental importance. Given the above-mentioned, we hypothesized that the local administration of mp-HNK1 may stimulate the guided regeneration of motor axons following ventral root avulsion and reimplantation. Additionally, we used a systemic treatment approach with UA, an HNK-1 mimetic, administered orally for 14 days to assess its neuroprotective role in the context of a proximal lesion. Herein, we show that the local application of mp-HNK-1 following root reimplantation is neuroprotective and allows for guided regrowth of motor axons towards the target muscle. Also, systemic delivery of UA resulted in long-term neuroprotection coupled with immunomodulation of astroglial and microglial reactions. Overall, UA decreased the density of glutamatergic inputs to the axotomized motoneurons, in turn, reducing excitotoxicity and contributing to an enhanced motor behavior recovery.

## Methods

### mp-HNK-1 synthesis

The mp-HNK-1 was synthesized with a ResPepSL automated synthesizer (Intavis) using Rink amide resin as a solid phase and Fmoc-protected amino acids (Carbolution) for coupling according to the method of Merrifield. Resin-coupled peptides were cleaved and deprotected with trifluoroacetic acid (Sigma Aldrich), followed by precipitation with tert-butyl methyl ether (Fisher Scientific) and further analysis and purification by preparative HPLC (Merck) (purity > 95%). The overall yield was 101 mg for HNK1-peptidomimetic. Lyophilized peptides (Lyovac GT2, Finn-Aqua) were stored at -20°C. Peptide stock solutions were prepared and used on the same day.

### Animals and experimental groups

For this study, 8-week-old female C57BL/6JUnib mice obtained from the Multidisciplinary Center for Biological Investigation (CEMIB/UNICAMP, Campinas, SP, Brazil) were used. The animals were maintained with food and water *ad libitum* and in 12/12 h light/dark cycles at a constant temperature of 23.0 ± 1.0 ºC, 50% relative humidity, in the Laboratory of Nerve Regeneration (LRN/UNICAMP, Campinas, SP, Brazil). Protocols concerning animal use and handling were approved by the Institutional Committee for Ethics in Animal Experimentation (Committee for Ethics in Animal Use-Institute of Biology-CEUA/IB/UNICAMP, Protocol number 5516-1/2020) and were performed following the guidelines of the Brazilian College for Animal Experimentation.

The mice were randomly divided into four experimental groups according to the surgical procedure and treatment they were submitted: Avulsion, Reimplantation, Reimplantation + mp-HNK-1 (mp-HNK-1 group), and Reimplantation + Ursolic Acid treatment (UA group). Mice from the different experimental groups were kept for 2 or 12 weeks, and processed for the different experiments, as detailed in Table 1.

**Table 1.** Number of mice per experimental group, techniques, and time points of analyses.

Experimental group/Technique	2 weeks	12 weeks	
	*Motoneuron survival and immunofluorescence*	*Motoneuron survival and immunofluorescence*	*Catwalk*
Avulsion	5	5	5
Reimplantation	5	5	5
mp-HNK-1	5	5	5
Ursolic Acid	5	5	5

### Surgical procedure and treatments

All animals were sedated and pre-anesthetized with a combination of xylazine hydrochloride (Anasedan®, 10 mg/kg, Sespo, Brazil) and ketamine hydrochloride (Dopalen®, 100 mg/kg, Sespo, Brazil) applied intraperitoneally. Mice were then positioned on a heated mat and kept under inhalation anesthesia with isoflurane (1-2%) throughout the surgery. A lubricating ophthalmic gel (Liposic, Carbomer 2 mg + Sorbitol 48.5 mg; Bausch + Lomb) was applied to the eyes. 

The animals were shaved in the middle region of the back and, in the absence of the toe-pinch reflex, an incision parallel to the spine was made to the skin. The paravertebral musculature was removed to reach L1 and L2 vertebrae, and the laminectomy was performed unilaterally to expose the lumbar intumescence. The dura mater was opened through a longitudinal incision, and, after dissection of the denticulate ligament, the spinal cord was carefully moved to allow the identification of the L4, L5, and L6 ventral roots, which were avulsed using an Nº 4 forceps under a surgical magnifying microscope (DF Vasconcellos®). 

To mice that belonged to the Avulsion group, the heterologous fibrin biopolymer (HFB) was applied to the lesion site with no root reattachment. The HFB is composed of three fractions that are homogenized and applied in sequence at the lesion site totaling a volume of 2.5 μL: (I) fibrinogen cryoprecipitate derived from the blood of *Bubalus bubalis* (1 μL), (II) calcium chloride diluent (1 μL), and (III) gyroxin, a thrombin-like enzyme from the *Crotalus durissus terrificus* snake (0.5 μL) [48-51]. The third fraction added is responsible for the polymerization process, allowing the coaptation and stabilization of the roots, which can be observed during the surgical process. HFB components and application formulas are listed in its patent (BR1020140114327) and were kindly provided by the Center for the Study of Venoms and Venomous Animals (CEVAP/UNESP, Brazil).

**Figure 1.**
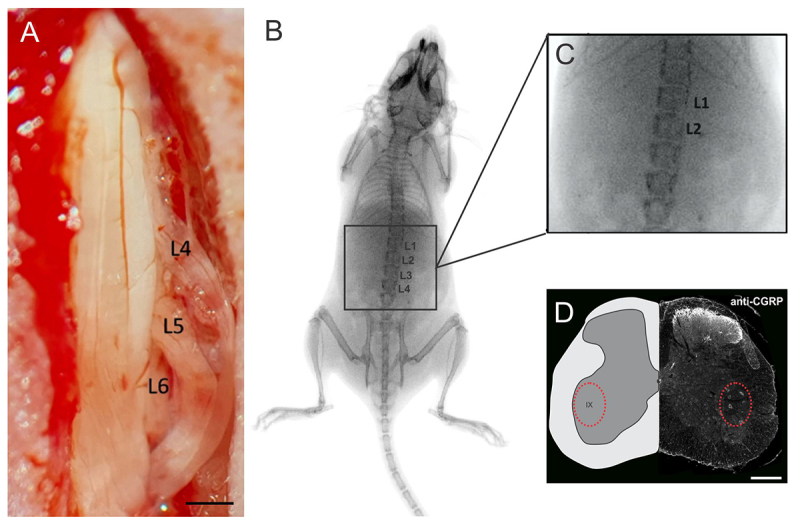
Surgical approach for ventral root avulsion and reimplantation. **(A)** View of the surgical window showing avulsed and reimplanted ventral roots. The L4, L5, and L6 ventral spinal roots were reconnected to the spinal cord surface using the HFB. Scale bar = 1 mm. **(B)** Radiograph of a mouse after surgery, showing the vertebral column and detailed at the **(C)** inset the L1 and L2 vertebrae that underwent hemilaminectomy (right side) to expose the lumbar intumescence and the ventral roots. **(D)** Transverse section of the lumbar spinal cord depicting the CGRP-positive motoneurons localized in the dorsolateral nucleus of the lamina IX of Rexed (red dotted circle). Scale bar = 500 µm.

In the mice belonging to the experimental groups that received reimplantation, the roots were repositioned to the spinal cord surface as close as possible to their original location, and HFB was applied for coaptation [52] (Figure 1). 

In the mp-HNK-1 group, 100 ng of the glycomimetic mp-HNK-1 was applied homogenized to the HFB, which acted as a scaffold for the peptide [39, 53].

Finally, the mice from UA group were submitted to ventral root avulsion and reimplantation procedure as described above and treated with UA (Sigma-Aldrich® - U6753) at a dose of 200 mg/kg, diluted with 1% Dimethylsulfoxide (DMSO) in saline solution. The treatment was performed via gavage, daily, for two weeks. The dose was chosen based on a literature review on the effectiveness of UA and DMSO concentrations [26, 45, 47].

After the surgical procedures, the spinal cord and ventral roots remained in their original position, and the musculature, fascia, and skin were sutured in layers. Mice were allowed to recover in a post-surgical environment, where they were kept warm for four hours. An analgesic (tramadol hydrochloride, 5 mg/kg) was used subcutaneously for three consecutive days after surgery. The animals were kept for a period of 2 or 12 weeks, according to the experimental group.

### Euthanasia

After the predetermined period of survival (2 or 12 weeks), the animals received an overdose of the combination of ketamine (Dopalen®, 300 mg/kg, Sespo, Brazil) and Xylazine (Anasedan®, 30 mg/kg, Sespo, Brazil). Immediately afterward, they were submitted to thoracotomy and transcardiac perfusion of the vascular system with saline solution (0.9% NaCl in sodium phosphate buffer; PB 0.1 M, pH 7.4). Tissue fixation was performed with 4% paraformaldehyde (PFA 4%) in 0.1 M PB. The lumbar intumescences were subsequently dissected and post-fixed in the same fixative solution at 4 ºC for 24 hours. Following, the specimens were cryopreserved by immersion in solutions with increasing concentrations of sucrose: 10%, 20%, and 30%, 12 hours each. Then, the spinal cords were included in Tissue-Tek® (Miles Inc., USA) and frozen at temperatures from -38º to -40 ºC. Subsequently, cross sections (12 µm thickness) of the lumbar spinal cords were obtained in a cryostat (HM525, Microm), and slides were kept at -20 ºC until use. 

### Neuronal survival

For the motoneuron counting, cross-sections of the lumbar spinal cord were stained with Toluidine Blue (0.05% in distilled water), dehydrated diaphanized, and mounted with Permount (Fisher Scientific) and coverslip. The slides were analyzed under the microscope (DM5500B, Leica Microsystems), and the motoneurons located in the dorsolateral nuclei at lamina IX of Rexed of the ipsi- and contralateral sides of the spinal cord were counted. Approximately 20 alternate slices spaced 240 μm apart were used, and the counts were corrected using the Abercrombie formula [54]:

N=nt/(t+d)

where N is the corrected number of counted neurons, n is the number of counted neurons, t is the distance between the counted sections, and d is the mean neuron diameter. As the difference in cell size significantly affects the number of cells, the d-value was calculated specifically for each experimental group (ipsilateral and contralateral). In this sense, the diameter of 15 motoneurons for each group was measured using the Image J software (version 1.33u) and the mean was calculated.

Subsequently, the ipsi/contralateral ratio was calculated, which corresponds to the neuronal survival rate, and the mean ± standard error was calculated for each experimental group.

### Immunofluorescence

The slides obtained in cryostat were climatized and washed in PB 0.01 M, followed by incubation with bovine serum albumin (BSA 3%) for one hour. Then, the primary antibodies (Table 2) were diluted in 1% BSA + 0.2% Triton in 0.1 M PB, and incubated overnight, in a humid chamber at 4 ºC. After, the slices were washed again with PB 0.01 M and incubated with the respective secondary antibodies in a humid chamber at room temperature for 45 minutes. The slides were finally washed in PB 0.001 M, mounted in Glycerol/PB 0.1 M (3:1), and kept at -20 ºC until use. For the immunofluorescence analysis, the ipsilateral and contralateral sides of the ventral horn of the spinal cord were observed and documented using a Leica DM 5500B fluorescence microscope, coupled to a Leica DFC 345FX camera, using the appropriate filters for cyanine 2 (Cy2, 492 nm - excitation and 510 - emission) or cyanine 3 (Cy3, 550 nm - excitation and 570 nm - emission). The imaging acquisition was standardized in terms of exposure time and camera gain for each antibody.

**Table 2.** Primary antibodies used for the immunofluorescence technique.

Antibody	Manufacturer	Code	Dilution
Anti-GFAP	Abcam^®^	ab7260	1:750
Anti-Iba-1	Wako^®^	019-19741	1:700
Anti-VGLUT 1	Synaptic Systems^®^	135303	1:1000
Anti-GAD65	Abcam^®^	ab26113	1:750
Anti-CGRP	Sigma Aldrich	C8198	1:1000

Three images per animal of each experimental group were obtained and proceeded to quantitative analysis through the integrated density of pixels (IDP) assessment, which represents the immunolabeling intensity. The integrated density of pixels was measured using Image J software (1.33u version, National Institutes of Health, USA) according to previous works [55, 56]. Briefly, 8-bit images were submitted to threshold segmentation, and the threshold value cutoff was set by image comparison with the RGB counterpart. The whole area of the picture at 20× was quantified. The IDP was acquired in the ipsi and contralateral sides of the spinal cord of each animal. The mean ± standard error of the mean for each experimental group was calculated.

### Walking track test (Catwalk platform)

The Catwalk System (Noldus Inc., The Netherlands) assessed the animals’ gait after injury for 12 weeks. The mice crossed the lighted glass walkway (100 cm long × 15 cm wide × 0.6 cm thick) in a dark room. A green fluorescent light illuminates the edge of the floor, distributed homogeneously along the path, enabling the intensification of the areas in which the animals’ paws meet the glass floor.

The illuminated footprint technology allows the detection of subtle pressure differences, enabling the evaluation of the animal’s body weight distribution on the paws. Data acquisition was performed using a high-speed camera (Fujinon DF6HA-1B, sampling rate of 100 frames per second), positioned below the platform, and the Catwalk software automatically classified the data. Footprints were collected once before and every four days after the surgical procedure, until the end of the experimental period (12 weeks). At each evaluation, three runs were recorded per animal. The parameters Stand, Swing, Max Contact Area, Max Contact Max Intensity, Max Contact At (%), and the print width and length of the paws were acquired. 

An average was computed for the preoperative time-point and for each subsequent time-point post-surgery within each experimental group and presented as mean ± standard error of the mean. The Sciatic Functional Index (SFI) was calculated using two parameters: the distance between the first and fifth toes (toe spread, TS - paw width) and the distance between the third toe and heel (print length, PL - paw length). These parameters were used to measure the footprints of the right hind paw (injured) and left hind paw (normal); the values were applied in the following formula, described by [57]:

SFI=118.9×((ETS-NTS)/NTS)-51.2×((EPL-NPL)/NPL)-7.5

(E = injured side, N = normal side)

### Statistical analysis

Data are presented as the mean ± standard error of the mean (SEM). For all analyses, GraphPad Prism 8 software was used. Neuronal survival and immunofluorescence data were evaluated using the one-way analysis of variance - ANOVA. The data from the functional analysis (Walking Track Test) were evaluated using the two-way analysis of variance - ANOVA. In both cases, Tukey’s post-test was performed. Significance levels of (*) p < 0.05, (**) p < 0.01, (***) p < 0.001, and (****) p < 0.0001 were assumed.

## Results

### Short and long-term effects of reimplantation and UA treatment on motoneuronal survival

**Figure 2.**
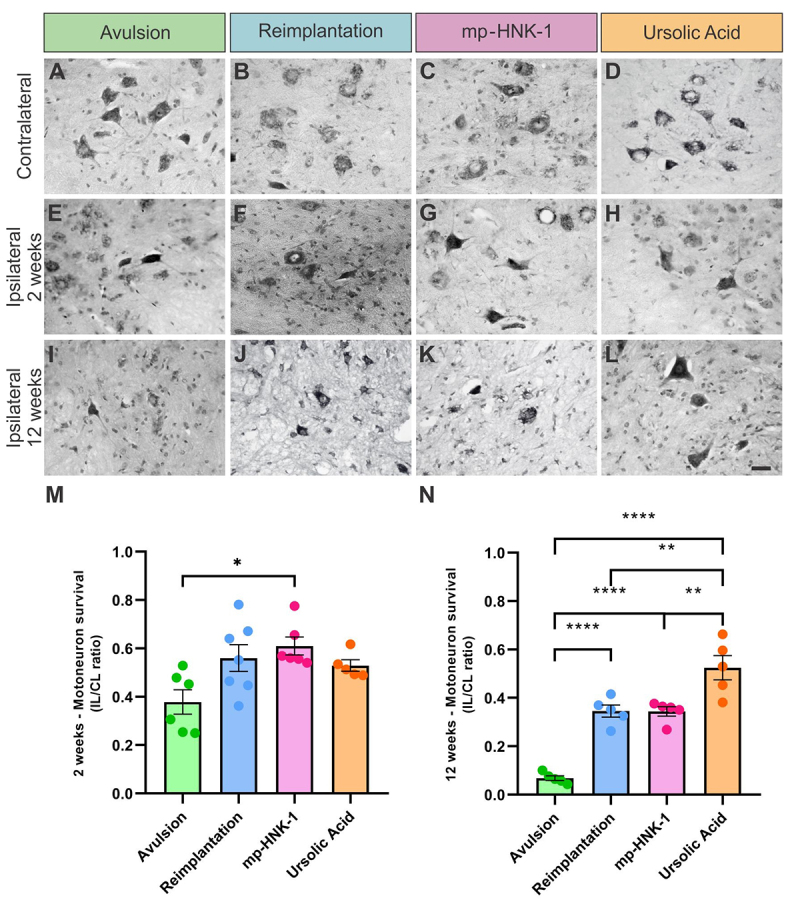
Motoneuron survival at 2 and 12 weeks after injury, in spinal cord cross-sections stained with toluidine blue. **(A-D)** Contralateral sides of the Avulsion, Reimplantation, mp-HNK-1, and UA groups, respectively. **(E-H)** Ipsilateral sides at two weeks after injury in the Avulsion, Reimplantation, mp-HNK-1, and UA groups, respectively. **(I-L)** Ipsilateral sides of the Avulsion, Reimplantation, mp-HNK-1, and UA groups, respectively. **(M and N)** Ipsi/contralateral ratio for the analysis of motoneuron survival at 2 and 12 weeks after injury, respectively. Long-term neuronal preservation was observed in the Reimplantation, mp-HNK-1, and UA groups, as compared to the Avulsion. Observe that the most beneficial effects were obtained in the UA group. One-way ANOVA followed by Tukey multiple comparisons test; *p < 0.05; **p < 0.01; ***p < 0.001. Data presented as mean ± SEM. Scale bar = 50 μm.

To evaluate the effectiveness of treatment with mp-HNK-1 and UA after avulsion and reimplantation with HFB, a comparison was made between them and the respective controls through the percentage of neuronal survival. The analyses were carried out 2 and 12 weeks (Figure 2) after injury.

It was possible to identify signs of degeneration of motor neurons on the ipsilateral sides of all experimental groups, being more severely affected in animals from the Avulsion group when compared to the Reimplantation groups, mp-HNK-1 and UA. Such observation translates into the mean value calculated for each experimental group. At the 2-weeks’ time-point, the mean ± SEM was: Avulsion = 0.36 ± 0.06, Reimplantation = 0.58 ± 0.06, mp-HNK-1 = 0.61 ± 0.04 and AU = 0.53 ± 0.02. Both Reimplantation and Reimplantation + mp-HNK displayed significant differences when compared to the Avulsion (*p < 0.05).

Long-term analysis showed that the neuronal loss is more pronounced 12 weeks after the lesion, especially in the Avulsion group (****p < 0.0001 compared to the other experimental groups). Reimplantation and mp-HNK-1, despite a slight decrease in neuronal death when compared with the two-week data, show a long-term neuroprotective effect. The UA group, however, stands out, demonstrating a neuronal survival rate equivalent to that found two weeks after injury, with significantly higher survival compared to the other experimental groups (****p < 0.0001 compared to the Avulsion group; and **p < 0.01 compared to Reimplantation and mp-HNK-1 groups). At 12 weeks after injury, mean neuronal survival values ± SEM: Avulsion = 0.07 ± 0.01, Reimplantation = 0.35 ± 0.03, mp-HNK-1 = 0.34 ± 0.02, UA= 0.52 ± 0.05

### Evaluation of microglial reaction and reactive astrogliosis

**Figure 3.**
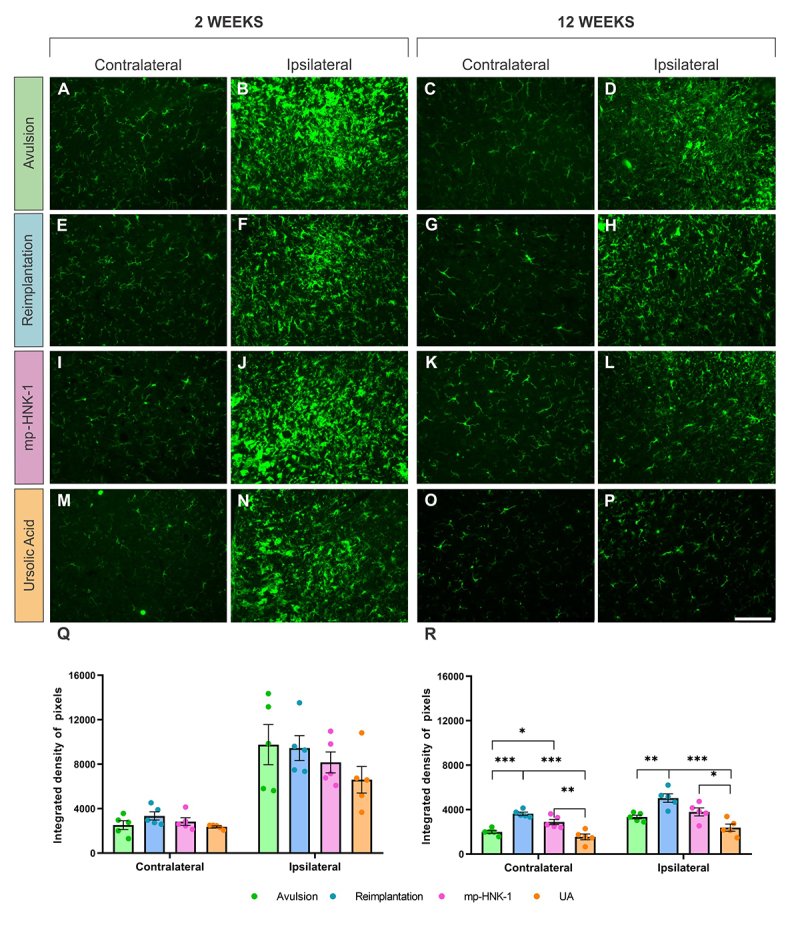
(A-D) Anti-Iba-1 immunostaining on the contra- and ipsilateral sides of the spinal cord 2 and 12 weeks after lesion in the Avulsion, (E-H)Reimplantation, (I-L) mp-HNK-1, and (M-P) AU groups. Graphs of the integrated density of pixels in the contra and ipsilateral sides in the different experimental groups at (Q) 2 and (R) 12 weeks after lesion. Intense microglial reactivity is observed on the ipsilateral side compared to the respective contralateral sides. There is a slight decrease in glial reactivity in the UA group at 12 weeks. One-way ANOVA, followed by Tukey multiple comparisons test; *p < 0.05; **p < 0.01; ***p < 0.001. Data presented as mean ± SEM. Scale bar = 100 µm.

To assess putative changes in the microglial reactivity after injury and treatment, we analyzed the immunoreactivity for the Iba-1 protein in the ventral horn of the lumbar spinal cord (L4-L6) through integrated density of pixels (IDP) quantification in the contra- and ipsilateral sides of the spinal cord (Figure 3). The comparative analysis two weeks after injury, although it showed an increase in the IDP on the ipsilateral sides of all experimental groups, such an increase was not significantly different among them. Contralateral IDP at two weeks, mean ± SEM: Avulsion = 2522 ± 394, Reimplantation = 3345 ± 371, mp-HNK-1 = 2838 ± 344 and UA = 2367 ± 82. Ipsilateral IDP at two weeks, mean ± SEM: Avulsion = 9760 ± 1810, Reimplantation = 9448 ± 1117, mp-HNK-1 = 9160 ± 946 and UA = 6604 ± 1192.

At 12 weeks, on the other hand, there was an increase in the IDP detected on the contralateral sides of the Reimplantation and mp-HNK-1 groups (***p < 0.001, and *p < 0.05, compared to the Avulsion) which indicated an interesting bilateral effect of the lesion on the microglial reaction, which was reduced with UA treatment (***p < 0.001 compared to Reimplantation and **p < 0.01 compared to mp-HNK-1). On the ipsilateral sides, at 12 weeks, although the overall IDP decreased with time among all experimental groups, still differences were detected with an increase in the IDP in the Reimplantation group compared to the Avulsion (**p < 0.01) and a decrease of such reaction by the treatment with UA (***p < 0.001 compared to the Reimplantation).

Contralateral IDP at 12 weeks, mean ± SEM: Avulsion = 1994 ± 138, Reimplantation = 3634 ± 147, mp-HNK-1 = 2885 ± 242 and UA = 1547 ± 264. Ipsilateral IDP at 12 weeks, mean ± SEM: Avulsion = 3333 ± 167, Reimplantation = 5039 ± 385, mp-HNK-1 = 3790 ± 361 and UA = 2378 ± 322.

**Figure 4.**
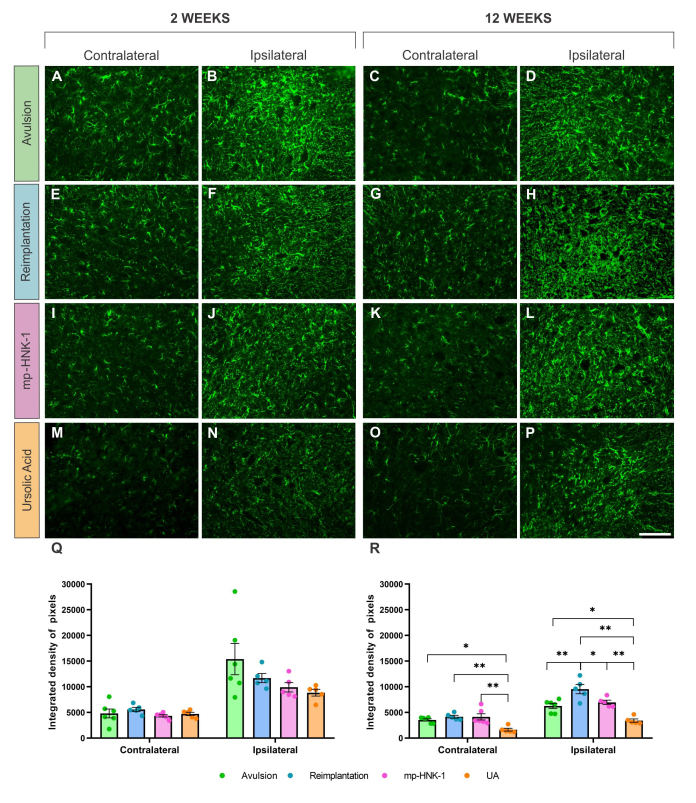
(A-D) Anti-GFAP immunostaining on the contra- and ipsilateral sides of the spinal cord 2 and 12 weeks after lesion in the Avulsion, (E-H) Reimplantation, (I-L) mp-HNK-1, and (M-P) AU groups. Graphs of the integrated density of pixels in the contra and ipsilateral sides in the different experimental groups at (Q) 2 and (R) 12 weeks after lesion. Intense reactivity is observed on the ipsilateral side compared to the respective contralateral sides. There is a decrease in astrogliosis at 12 weeks in mp-HNK-1 and UA groups. One-way ANOVA, followed by Tukey multiple comparisons test; *p < 0.05; **p < 0.01; Data presented as mean ± SEM. Scale bar = 100 µm.

To assess the reactive astrogliosis resulting from the lesion and the effects of the different treatments, an analysis of the immunoreactivity for the GFAP protein was performed in the ventral horn of the lumbar spinal cord (L4-L6) at 2 and 12 weeks after the injury (Figure 4). 

Quantitative evaluation of immunostaining consisted of obtaining the integrated density of pixels on the ipsilateral and contralateral sides of each group. An increase in GFAP expression was observed in the ipsilateral sides of all experimental groups when compared to their respective contralateral sides. Such an increase is expected since reactive astrogliosis is a characteristic consequence of the lesion.

Despite the increase in the ipsilateral side compared to the contralateral, no intergroup difference was detected at two weeks after injury. Contralateral IDP at two weeks, mean ± SEM: Avulsion = 4803 ± 862, Reimplantation = 5583 ± 411, mp-HNK-1 = 4329 ± 228 and UA = 4712 ± 334. Ipsilateral IDP at two weeks, mean ± SEM: Avulsion = 15392 ± 3049, Reimplantation = 11677 ± 876, mp-HNK-1 = 9903 ± 921 and UA = 8844 ± 658.

At 12 weeks, there was a general tendency of anti-GFAP immunostaining to decrease, but still displaying statistically higher staining in the contralateral sides of the Avulsion, Reimplantation, and mp-HNK-1, when compared to the UA group (respectively: *p < 0.05; **p < 0.01 and **p < 0.01). Contralateral IDP at 12 weeks, mean ± SEM: Avulsion = 3537 ± 226, Reimplantation = 4170 ± 277, mp-HNK-1 = 4280 ± 714 and UA = 1648 ± 275.

Differences were also detected in the ipsilateral sides, mostly by the increase of the astrogliosis by the Reimplantation (**p < 0.01 compared to the Avulsion) and the decrease by mp-HNK-1 (*p < 0.05) and UA (**p < 0.01) treatments that were combined with reimplantation. Ipsilateral IDP at 12 weeks, mean ± SEM: Avulsion = 6251 ± 500, Reimplantation = 9540 ± 911, mp-HNK-1 = 6968 ± 408 and UA = 3413 ± 319.

### Evaluation of glutamatergic and GABAergic synapses

**Figure 5.**
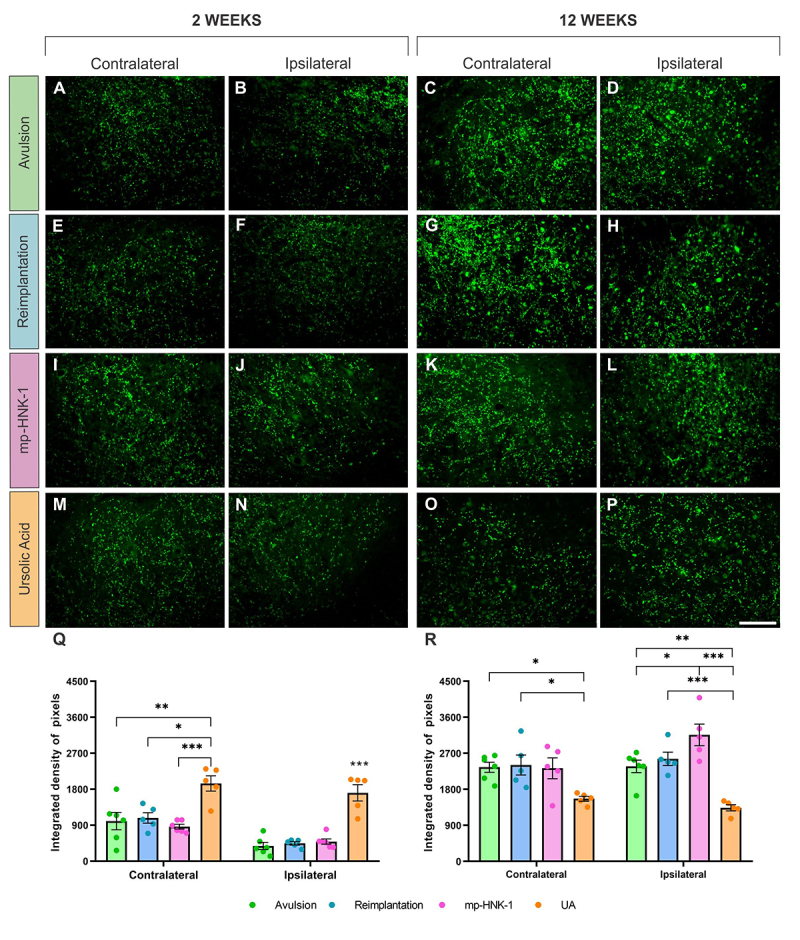
(A-D) Anti-VGLUT-1 immunostaining on the ipsilateral and contralateral sides of the lesion 2 and 12 weeks after the lesion in the Avulsion, (E-H) Reimplantation, (I-L) mp-HNK-1, and (M-P) AU groups. Graphs of the integrated density of pixels in the contra and ipsilateral sides in the different experimental groups at (Q) 2 and (R) 12 weeks after lesion. Acutely, a greater intensity of excitatory vesicles is observed in the UA group at two weeks, which decreases in the long term. One-way ANOVA, followed by Tukey multiple comparisons test; *p < 0.05; **p < 0.01; ***p < 0.001. Data presented as mean ± SEM. Scale bar = 100 µm.

**Figure 6.**
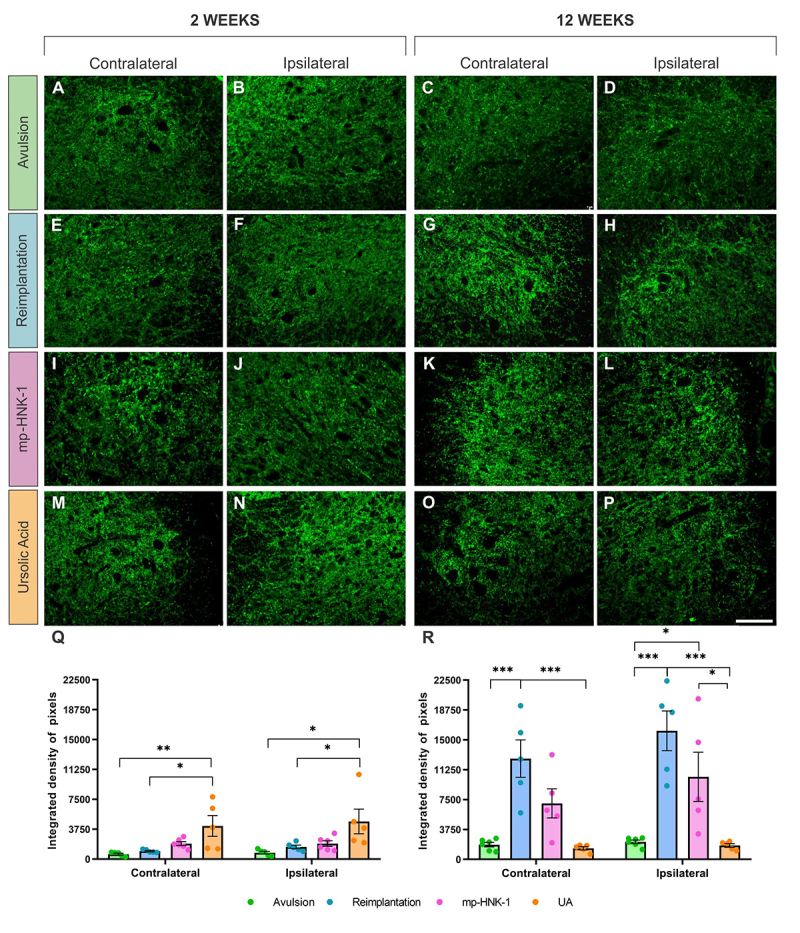
(A-D) Anti-GAD-65 immunostaining on the ipsilateral and contralateral sides of the lesion 2 and 12 weeks after the lesion in the Avulsion, (E-H) Reimplantation, (I-L) mp-HNK-1, and (M-P) AU groups. Graphs of the integrated density of pixels in the contra and ipsilateral sides in the different experimental groups at (Q) 2 and (R) 12 weeks after lesion. A greater intensity of inhibitory vesicles is observed in the UA group in the acute analysis, which was decreased by the 12th week. One-way ANOVA, followed by Tukey multiple comparisons test; *p < 0.05; **p < 0.01; ***p < 0.001. Data presented as mean ± SEM. Scale bar = 100 µm.

To assess changes in glutamatergic and GABAergic synapses resulting from the injury and the different associated therapies, immunoreactivity analyses were performed using anti-VGLUT-1 (Figure 5) and anti-GAD-65 (Figure 6) antibodies. For quantitative evaluation, the integrated density of pixels of the ipsilateral and contralateral sides was obtained for each experimental group.

VGLUT-1 is a glutamate transporter found in the membrane of glutamatergic synaptic vesicles and two weeks after the lesion there was a decrease in the glutamatergic inputs at lamina IX in the ipsilateral side of all experimental groups, when compared to their contralateral sides, except for the UA group, which showed increased VGLUT-1 immunostaining (**p < 0.01 compares to Avulsion, *p < 0.05 to Reimplantation, and ***p < 0.001 to mp-HNK-1). Contralateral IDP at 2 weeks, mean ± SEM: Avulsion = 1003 ± 216, Reimplantation = 1082 ± 132, mp-HNK-1 = 864 ± 59 and AU = 1944 ± 191.

Separated analysis of the IDP on the ipsilateral sides two weeks after the lesion still showed greater VGLUT1^+^ preservation in the UA when compared to other lesioned groups (***p < 0.001). Ipsilateral IDP ± SEM: Avulsion = 379 ± 90, Reimplantation = 446 ± 43, mp-HNK-1 = 491 ± 67 and UA = 1709 ± 204.

When analyzed 12 weeks after the injury, the tendency was for contralateral IDP to increase in all experimental groups except for the UA (*p < 0.05 compared to Avulsion and Reimplantation). Contralateral IDP at 12 weeks, mean ± SEM: Avulsion = 2352 ± 126, Reimplantation = 2407 ± 250, mp-HNK-1 = 2326 ± 260 and UA = 1565 ± 62.

The analysis of the IDP on the ipsilateral sides identified significant differences between UA and Avulsion (**p < 0.01), Reimplantation (***p < 0.001), and mp-HNK-1 (***p < 0.001) groups. Mean ipsilateral IDP ± SEM: Avulsion = 2373 ± 156, Reimplantation = 2560 ± 167, mp-HNK-1 = 3160 ± 270 and UA = 1338 ± 76, 

Regarding GAD65 (Figure 6), two weeks after the lesion, separated analysis of the IDP on the ipsilateral sides two weeks after lesion, however, depicted an increase in the GAD65^+^ fibers in the UA group compared to Avulsion and Reimplantation (*p < 0.05). Mean contralateral IDP ± SEM: Avulsion = 1072 ± 303, Reimplantation = 1531 ± 230, mp-HNK-1 = 1944 ± 346 and UA = 4722 ± 1551. Significant differences in GAD65 immunostaining in the contralateral sides were also depicted in the UA group when compared with the Avulsion and the Reimplantation groups (*p < 0.05), reaffirming its bilateral synaptic-protective action.

In the long term, 12 weeks after the lesion, analysis of the ipsilateral sides depicted an increase in the anti-GAD65 immunolabeling in the Reimplantation group compared to the Avulsion and the UA (***p < 0.001) groups, and in the mp-HNK-1 compared to the Avulsion and the UA (*p < 0.05) groups. Mean ipsilateral IDP ± SEM: Avulsion = 2196 ± 225, Reimplantation = 16133 ± 2499, mp-HNK-1 = 10356 ± 3095 and UA = 1744 ± 232. The reimplantation also showed increased GAD65 immunostaining in the contralateral sides when compared to the Avulsion (***p < 0.001) and the UA (***p < 0.01) groups. Mean contralateral IDP ± SEM: Avulsion = 1854 ± 283, Reimplantation = 12651 ± 2346, mp-HNK-1 = 7029 ± 1824 and UA = 1399 ± 206.

### Functional recovery evaluation

The Catwalk system provides several gait parameters, enabling the tracking of functional recovery after injury. We seek to compare the functional recovery rates achieved by the animals of the different experimental groups over 12 weeks post-surgical using several parameters mainly subdivided into time or spatial-related. 

**Figure 7.**
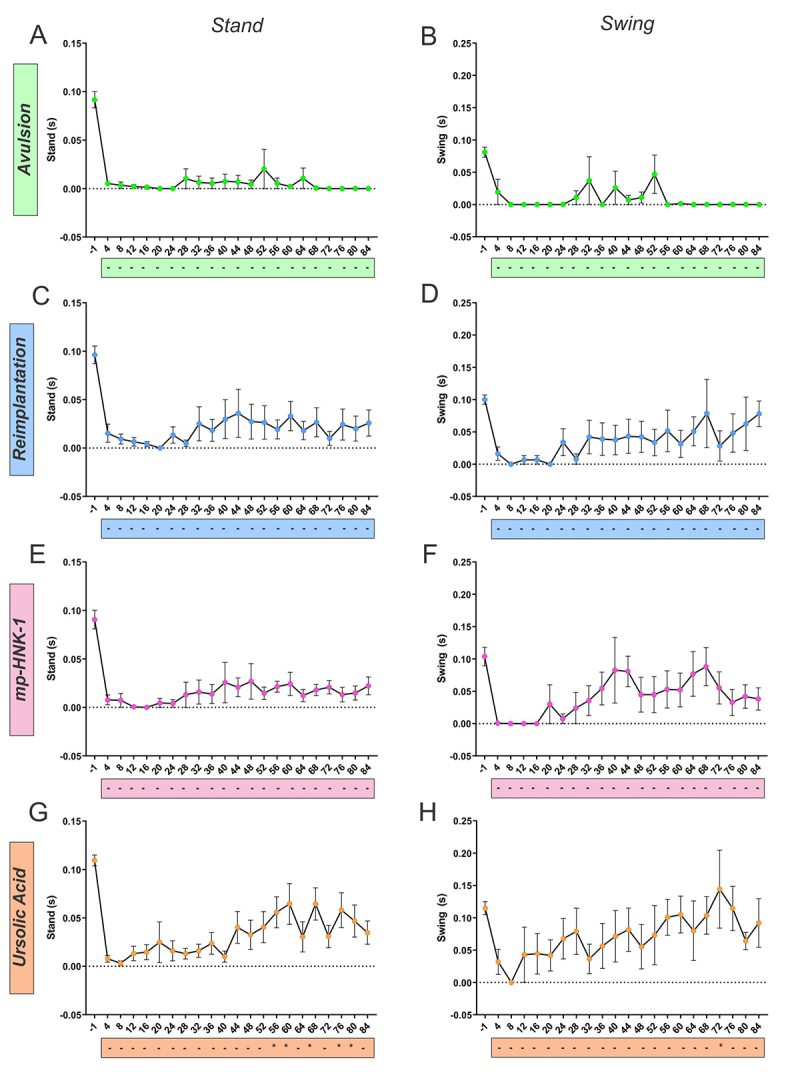
Catwalk gait analysis for the stand and swing parameters in **(A and B)** Avulsion, **(C and D)** Reimplantation, **(E and F)** mp-HNK-1, and **(G and H)** Ursolic Acid groups. The recovery in each group was analyzed by comparison against the first training session (day 4). Only the UA-treated group presented significant recovery at certain time points as indicated by the asterisks in the boxes below the graphs. Two-way ANOVA, followed by Tukey multiple comparisons test. Data presented as mean ± SEM.

The time-related parameters are here represented by the Stand and Swing, measured in seconds. The step cycle of a paw is composed of a Stand phase, the time interval in which such a limb is in contact with the glass plate, and a Swing phase, the duration of no contact of the paw with the glass (Figure 7). Related to the stand, in general, in the Avulsion group, the mice tend to neglect the ipsilateral paw, which did not touch the glass floor up to the 24th day after the lesion, and with minimum contact afterward. The reimplantation leads to the acceleration of the ipsilateral paw use and an overall intensification of the stand phase. The local administration of mp-HNK-1 did not surplus to the effect of the reimplantation. However, treatment with Ursolic Acid is related to more effective use of the ipsilateral paw by the 12th day post-lesion and forward, with a standing value that reaches 60% of the baseline in the 60th day after the lesion.

When analyzing the swing, it was also possible to detect that the Avulsion group exhibited the minimum value throughout the experiment, and no significant differences were detected among the Reimplantation, mp-HNK-1, and Ursolic Acid groups.

**Figure 8.**
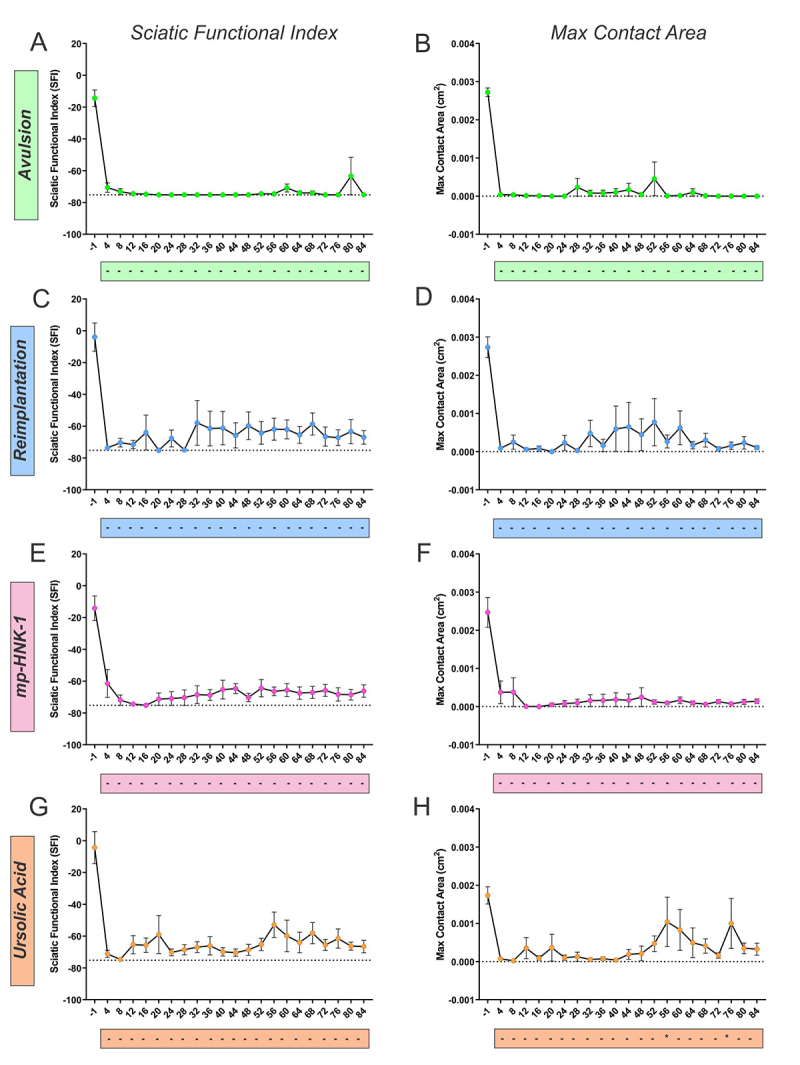
(A and B) Catwalk gait analysis for the SFI and Max Contact Area parameters in Avulsion, (C and D) Reimplantation, (E and F) mp-HNK-1, and (G and H) Ursolic Acid groups. The recovery in each group was analyzed by comparison against the first training session (day 4). Only the UA-treated group presented significant recovery at certain time points as indicated by the asterisks in the boxes below the graphs. Two-way ANOVA, followed by Tukey multiple comparisons test. Data presented as mean ± SEM.

**Figure 9.**
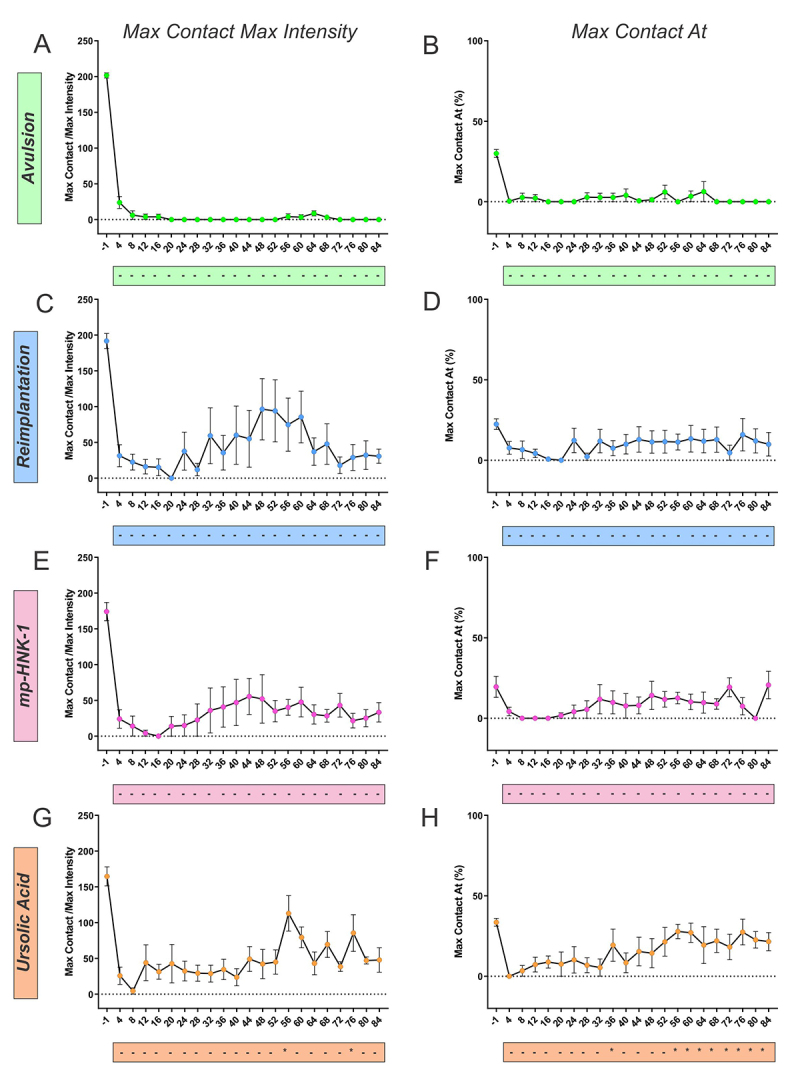
(A and B) Catwalk gait analysis for the Max Contact/Max Intensity and Max Contact At (%) parameters in Avulsion, (C and D) Reimplantation, (E and F) mp-HNK-1, and (G and H) Ursolic Acid groups.. The recovery in each group was analyzed by comparison against the first training session (day 4). Only the UA-treated group presented significant recovery at certain time points as indicated by the asterisks in the box below the graphs. Two-way ANOVA, followed by Tukey multiple comparisons test. Data presented as mean ± SEM.

The spatial-related category comprehends parameters that involve measurements of the dimensions of the paw or the pressure that the paw makes on the glass floor. Among the parameters, the print length and print width of the ipsi- and contralateral paws were used to calculate the Sciatic Functional Index (SFI); the Max Contact Area of the paw (Figure 8) was also used, as well as the Max Contact/Max Intensity, and the Max Contact At (Figure 9).

The SFI value in uninjured animals is around 0 and tends to decrease after lesion, up to -75.2, increasing with recovery. In the Avulsion group mice did not show SFI recovery, and the experimental groups in which reimplantation was done demonstrated a small recovery. The Max Contact Area, on the other hand, is the maximum area of a paw that meets the glass plate, and it was also greatly decreased by the Avulsion and minimally re-established by the reimplantation. The treatment with Ursolic Acid demonstrated effects by the increase in such parameters on days 56 and 76 after the lesion.

The Max Contact/Max Intensity parameter is the Maximum Intensity at the Max Contact of a paw and ranges from 0 to 255. It allows the assessment of weight bearing on the limb, an indicator that correlates with nociception. A sudden drop in the pressure exerted by the ipsilateral paw is observed in all experimental groups after the lesion. Over time, despite the groups showing different degrees of improvement, the UA group showed statistically significant functional recovery on days 56 and 76 after injury. The Max Contact At represents the greatest weight‐bearing exerted during gait to the total time the paw is in contact with the platform. Such parameter greatly decreases with Avulsion and minimally recovers with Reimplantation, but Ursolic Acid treatment showed beneficial effects in the recovery starting from day 56 after lesion and forward.

## Discussion

Proximal lesions that occur in the peripheral and central nervous system (PNS and CNS, respectively) interface are debilitating once they impair motor and sensory function, with limited spontaneous functional recovery [6]. The development of animal models is useful for evaluating the mechanisms of motoneuron death, also allowing the prospection of new therapeutic approaches, aiming at the study of neuroprotection, preservation of spinal synaptic circuitry, and functional recovery after radicular avulsion.

Pioneering studies using animal models for ventral root lesions were carried out extensively in rats [2, 8, 58], and the adaptation and standardization of the microsurgery in mice is extremely advantageous, given the easy access and maintenance of animals in vivariums, in addition to the range of transgenic mice generated in recent years. In our laboratory, we standardized the ventral root crush in mice [55], and then proceeded to standardize a model for ventral root avulsion and reimplantation in mice, as described herein. 

By comparatively analyzing the long-term motoneuron survival in rats and mice after avulsion, we observed that the avulsion tended to have a greater impact on motoneurons in mice than in rats, as we detected 6% motoneuron survival 12 weeks after avulsion, in comparison with 30% described for rats in previous studies [48, 59].

Similarly, reimplantation of avulsed roots is known to have a neuroprotective effect, leading to the preservation of up to 60% of spinal motoneurons in rats [8, 48]. Such a neuroprotective effect is also observed in the mice model, which detected the preservation of approximately 35% of motoneurons 12 weeks after injury.

To boost the neuroprotective effect of reimplantation, several adjuvant therapeutic approaches have been associated with reimplantation, such as the administration of neuroprotective drugs and stem cell therapy [3, 59]. 

In this sense, HNK-1 is a glycan that has important functions in the nervous system development and plasticity [21, 22, 26, 29]. Due to its importance, several studies have been surveying its effects on various pathological conditions mainly by two approaches: the use of synthetic peptides that mimic the HNK-1 glycan [34, 39], or mimetic substances of HNK-1 which are naturally found in plants, for example, like the ursolic acid [43-45]. In the present study, we compared the effects of two different approaches combined with the reimplantation: the in situ application of 100 ng mp-HNK-1, or systemic treatment with ursolic acid.

Therefore, two weeks after injury, the mp-HNK-1 and UA groups showed a motoneuron survival rate that did not surpass the neuroprotective effect of the reimplantation. In the long-term, on the other hand, it was evident that UA treatment prevented the secondary loss of motoneurons, keeping the survival rate of up to 52% of the motoneurons, which was statistically the same as that found at two weeks. These data corroborate with those already described. In a model of ischemic injury in mice, UA exerted a neuroprotective effect, mainly due to its antioxidant and anti-inflammatory properties [42, 60].

In addition to motoneuron death, ventral root lesions also raise several retrograde changes in the spinal cord microenvironment, such as reactive gliosis and synaptic detachment [61]. Microglial cells are the first responders to any pathological event that reaches the parenchyma of the nervous system. These cells are the resident immune cells of the CNS, constantly scanning the parenchyma and rapidly reacting to any sign of stress [62]. In the acute phase, immune activation is necessary to promote the elimination of cellular debris, leading to the resolution of the inflammatory reaction. However, if they remain chronically activated, such cells can release pro-inflammatory molecules, resulting in greater tissue damage and potentially contributing to neurodegeneration [63]. The challenge is to optimize the immune responses, providing new strategies that will compensate for the tissue loss caused by the injury [63].

Herein, microglial reaction was assessed at 2 and 12 weeks after the lesion by Iba-1 immunolabeling. In the acute phase, intense microglial reactivity was detected on ipsilateral sides in all experimental groups. This result was expected, as hypertrophic microglial cells are observed in the region ipsilateral to the lesion, especially in close contact with avulsed motoneurons [64]. However, in the long-term analysis, residual microglial activation persisted in the reimplantation and mp-HNK-1 groups, and UA treatment led to a significant reduction in Iba-1 expression, indicating a decrease in microglial reactivity. Honarvar et al. [65] and Zhang et al. [66] described similar results, demonstrating that UA supplementation in a model of experimental autoimmune encephalomyelitis (EAE) led to a reduction in microglial cell activation.

When comparing the microglial reactivity obtained in the present work with that described in experimental models in rats, it was noted that, for rats, there was no difference in microglial reactivity between the avulsion and reimplantation groups [48, 67]. This differs from the data obtained in the present study, which demonstrated a significant increase in the microglial reaction 12 weeks after injury and reimplantation. 

Astrocytes are also known to respond to any insults that affect the CNS through a process called astrogliosis [68]. Chronically activated astrocytes are scar-forming and considered a major obstacle to axonal regeneration after injury. Therefore, modulating reactive astrocytes may contribute to the control of neuroinflammation and may reveal new molecular targets to stimulate repair processes [69, 70]. 

In this context, when analyzing reactive astrogliosis in the different experimental groups two weeks after injury, we observed an increase in GFAP immunostaining on the ipsilateral side of all experimental groups. Therefore, at 12 weeks, there was a significant reduction in astrogliosis in the UA group compared with the other groups. Sahu [26] also detected a decrease in GFAP expression in UA-treated mice after crushing T7-T9 roots, indicating possible antioxidant and anti-inflammatory effects, in parallel with the reduction of microglia. 

In the groups with 12 weeks of survival, a significant increase in astroglial reaction was observed in the reimplantation group compared to that in the avulsion group. This result differs from that obtained in rats by Barbizan [48], who demonstrated a significant reduction in reactive astrogliosis in the group with reimplantation and HFB. Similarly, the mp-HNK-1 also affects astrocytes, as previously described by Pedrazzi [71], highlighting that the peptide has pro-inflammatory activity in those cells.

The results described so far suggest that the different experimental models, mouse or rat, may present important differences in immunomodulation and regenerative responses. This finding suggests that, in the experimental model in mice, the overall glial reaction is more intense and tends to become chronic, which can partially explain the comparatively reduced performance of reimplantation in neuroprotection. Indeed, the pathology of spinal cord injury in rats and mice is known to be distinct [72], and differences in inflammatory responses have been observed even between mouse strains [73, 74].

Following the glial reaction, there is an extensive synaptic change in the axotomized motoneuron. It is known that proximal axotomy reduces the number of terminals that establish synaptic contacts with lesioned motoneurons. This reduction is more evident in excitatory terminals [75], and synaptic changes are also associated with a neuroinflammatory response, where microglia can often be seen adjacent to the motoneuron soma and major dendrites, removing synaptic terminals from neuronal membranes [76-78].

In this sense, it was possible to observe, two weeks after the lesion an intense reduction in VGLUT-1 positive terminals around the axotomized motoneurons in the ipsilateral sides of all experimental groups, except for the UA group, which showed remarkably more intense immunostaining both in the contralateral and ipsilateral sides of the spinal cord, demonstrating a bilateral effect of the injury and treatment.

On the other hand, the accumulation of glutamate can be putatively deleterious, owing to the possibility of excitotoxicity [79]. Therefore, in the long term, the glutamatergic inputs in the UA group remained stable over time and did not enhance, as depicted in other experimental groups, reducing the possibility of glutamate-mediated excitotoxicity.

The same pattern of acute increase, followed by synaptic remodeling in the long term, was found for GAD65 positive synapses, indicating the synaptoprotective effect of UA, but not the locally applied mp-HNK-1, in both glutamatergic and GABAergic inputs. These results reinforce the potential preferential elimination of glutamatergic synapses after axotomy, an autonomous cellular mechanism that differentially affects GABA/glycinergic and glutamatergic synapses, and is influenced by local glial reaction [56, 67, 76].

As a complement to the histological findings, analysis of functional recovery showed that there was no spontaneous recovery of the hind paws after avulsion. Reimplantation combined with UA treatment resulted in the partial functional recovery of indicators correlated with nociception and motor function. For instance, the stand value was closer to the baseline measurement in the UA group, and the ipsilateral paw exerted greater pressure intensity. In addition to the longer duration of the paw support phase and greater pressure, mice treated with UA also showed greater paw area in maximal pressure contact with the platform which demonstrated minimal recovery of reinnervation of the distal limb muscles, depicted by the toe abduction. On the other hand, animals treated locally with mp-HNK-1 did not show significant functional improvement regarding Stand, Max Contact Intensity, and Max Contact Area. 

Corroborating with our data, Sahu [26] also reports that oral administration of UA after spinal cord injury improved motor function recovery as well as axonal growth and decreased astrogliosis in mice. 

## Conclusion

In summary, the single local application of mp-HNK-1 did not demonstrate long-term neuroprotective effects nor significant synaptic changes and functional improvements. One hypothesis for this result is that the time of action of the locally applied mp-HNK-1 was short, not reaching the peak of expression for its ligands [80], and thus was unable to maintain the beneficial response in the long term. An indicator in favor of this hypothesis is the performance demonstrated by systemic treatment with AU, an HNK-1 mimetic, for 14 days. Therefore, this approach revealed a long-term neuroprotective effect, which was preceded by an acute effect on the spinal circuitry, acting on the maintenance of glutamatergic and GABAergic inputs. These findings correlated with the better performance of the group treated with UA in the functional recovery analyses.

## References

[B1] Carlstedt T (2007). Central nerve plexus injury. Imperial College Press.

[B2] Carlstedt T (2009). Nerve root replantation. Neurosurg Clin N Am.

[B3] Carlstedt T (2016). New Treatments for Spinal Nerve Root Avulsion Injury. Front Neurol.

[B4] Carlstedt T, Havton L (2012). The longitudinal spinal cord injury: lessons from intraspinal plexus, cauda equina and medullary conus lesions. Handb Clin Neurol.

[B5] Carlstedt T, James N, Risling M (2017). Surgical reconstruction of spinal cord circuit provides functional return in humans. Neural Regen Res.

[B6] Koliatsos VE, Loliatsos MD, Willian LP, Pardo CA, Price DL (1994). Ventral root avulsion: an experimental model of death of adult motor neurons. J Comp Neurol.

[B7] Carlstedt T, Grane P, Hallin RG, Norén G (1995). Return of function after spinal cord implantation of avulsed spinal nerve roots. Lancet.

[B8] Carlstedt T, Linda H, Cullheim S, Risling M (1986). Reinnervation of hind limb muscles after ventral root avulsion and implantation in the lumbar spinal cord of the adult rat. Acta Physiol Scand.

[B9] Eggers R, Tannemaat MR, de Winter F, Malessy MJA, Verhaagen J (2016). Clinical and neurobiological advances in promoting regeneration of the ventral root avulsion lesion. Eur J Neurosci.

[B10] Gordon T, Sulaiman O, Boyd JG (2003). Experimental strategies to promote functional recovery after peripheral nerve injuries. J Peripher Nerv Syst.

[B11] Gordon T, Yang JF, Ayer K, Stein RB, Tyreman N (1993). Recovery potential of muscle after partial denervation: a comparison between rats and humans. Brain Res Bull.

[B12] Barbizan R, Castro MV, Rodrigues AC, Barraviera B, Ferreira RS, Oliveira ALR (2013). Motor recovery and synaptic preservation after ventral root avulsion and repair with a fibrin sealant derived from snake venom. PLoS One.

[B13] Carlstedt T (1993). Functional recovery after ventral root avulsion and implantation in the spinal cord. Clin Neurol Neurosurg.

[B14] Cullheim S, Carlstedt T, Linda H, Risling M, Ulfhake B (1989). Motoneurons reinnervate skeletal muscle after ventral root implantation into the spinal cord of the cat. Neuroscience.

[B15] Hallin RG, Carlstedt T, Nilsson-Remahl I, Risling M (1999). Spinal cord implantation of avulsed ventral roots in primates; correlation between restored motor function and morphology. Exp Brain Res.

[B16] Ohlsson M, Hernán J, Christe KL Havton LA (2013). Long-term effects of a lumbosacral ventral root avulsion injury on axotomized motor neurons and avulsed ventral roots in a non-human primate model of cauda equina injury. Neuroscience.

[B17] Cartarozzi LP, Rieder P, Bai X, Scheller A, Oliveira ALR, Kirchhoff F (2018). In vivo two-photon imaging of motoneurons and adjacent glia in the ventral spinal cord.. J Neurosci Methods.

[B18] Gurumurthy CB, Lloyd KCK (2019). Generating mouse models for biomedical research: technological advances. Dis Model Mech.

[B19] Abo T, Balch CM (1981). A differentiation antigen of human NK and K cells identified by a monoclonal antibody (HNK-1). J Immunol.

[B20] Bronner-Fraser M (1987). Perturbation of cranial neural crest migration by the HNK-1 antibody. Dev Biol.

[B21] Kizuka Y, Oka S (2012). Regulated expression and neural functions of human natural killer-1 (HNK-1) carbohydrate. Cell Mol Life Sci.

[B22] Morise J, Takematsu H, Oka S (2017). The role of human natural killer-1 (HNK-1) carbohydrate in neuronal plasticity and disease. Biochim Biophys Acta Gen Subj.

[B23] Morita I, Kakuda S, Takeuchi Y, Itoh S, Kawasaki N, Kizuka Y, Kawasaki T, Oka S (2009). HNK-1 glyco-epitope regulates the stability of the glutamate receptor subunit GluR2 on the neuronal cell surface. J Biol Chem.

[B24] Nagase T, Sanai Y, Nakamura S, Asato H, Harii K, Osumi N (2003). Roles of HNK-1 carbohydrate epitope and its synthetic glucuronyltransferase genes on migration of rat neural crest cells. J Anat.

[B25] Nakamura A, Morise J, Yabuno-Nakagawa K, Hashimoto Y, Takematsu H, Oka S (2019). Site-specific HNK-1 epitope on alternatively spliced fibronectin type-III repeats in tenascin-C promotes neurite outgrowth of hippocampal neurons through contactin-1. PLoS One.

[B26] Sahu S, Li R, Kadeyala PK, Liu S, Schachner M (2018). The human natural killer-1 (HNK-1) glycan mimetic ursolic acid promotes functional recovery after spinal cord injury in mouse. J Nutr Biochem.

[B27] Bronner-Fraser M (1986). Analysis of the early stages of trunk neural crest migration in avian embryos using monoclonal antibody HNK-1. Dev Biol.

[B28] Castillo G, Kleene R, Schachener M, Loers G, Torda AE (2021). Proteins Binding to the Carbohydrate HNK-1: Common Origins?. Int J Mol Sci.

[B29] Eberhardt K, Irintchev A, Al-Majed AA, Simova O, Brushart TM, Gordon T, Schachner M (2006). BDNF/TrkB signaling regulates HNK-1 carbohydrate expression in regenerating motor nerves and promotes functional recovery after peripheral nerve repair. Exp Neurol.

[B30] Martini R, Schachner M, Brushart TM (1994). The L2/HNK-1 carbohydrate is preferentially expressed by previously motor axon-associated Schwann cells in reinnervated peripheral nerves. J Neurosci.

[B31] Gurwitz D (2017). Peptide Mimetics: Fast-Forward Look. Drug Dev Res.

[B32] Loers G, Liao Y, Hu C, Xue W, Shen H, Zhao W, Schachner M (2019). Identification and characterization of synthetic chondroitin-4-sulfate binding peptides in neuronal functions. Sci Rep.

[B33] Martínez-Villaluenga C, Hernández-Ledesma B (2020). Peptides for Health Benefits 2019. Int J Mol Sci.

[B34] Simon-Haldi M, Mantei N, Franke J, Voshol H, Schachner M (2002). Identification of a peptide mimic of the L2/HNK-1 carbohydrate epitope. J Neurochem.

[B35] Vagner J, Qu H, Hruby VJ (2008). Peptidomimetics, a synthetic tool of drug discovery. Curr Opin Cheml Biol.

[B36] Haggag YA (2018). Peptides as Drug Candidates - Limitations and Recent Development Perspectives. Biomed J Sci Tech Res.

[B37] Latham PW (1999). Therapeutic peptides revisited. Nat Biotechnol.

[B38] Sable R, Parajuli P, Jois S (2017). Peptides, Peptidomimetics, and Polypeptides from Marine Sources: A Wealth of Natural Sources for Pharmaceutical Applications. Mar Drugs.

[B39] Simova O, Irintchev A, Mehanna A, Liu J, Dihné M, Bachle D, Loers NSG, Schachner M (2006). Carbohydrate mimics promote functional recovery after peripheral nerve repair. Ann Neurol.

[B40] Ezra M, Bushman J, Shreiber D, Schachner M, Kohn J (2016). Porous and Nonporous Nerve Conduits: The Effects of a Hydrogel Luminal Filler With and Without a Neurite-Promoting Moiety. Tissue Eng Part A.

[B41] Stockwell BR (2004). Exploring biology with small organic molecules. Nature.

[B42] Wang Y, Li L, Deng S, Liu F, He Z (2018). Ursolic Acid Ameliorates Inflammation in Cerebral Ischemia and Reperfusion Injury Possibly via High Mobility Group Box 1/Toll-Like Receptor 4/NFκB Pathway. Front Neurol.

[B43] Jäger S, Trojan H, Kopp T, Laszczyk MN, Scheffler A (2009). Pentacyclic Triterpene Distribution in Various Plants - Rich Sources for a New Group of Multi-Potent Plant Extracts. Molecules.

[B44] Woźniak Ł, Skąpska S, Marszałek K (2015). Ursolic Acid--A Pentacyclic Triterpenoid with a Wide Spectrum of Pharmacological Activities. Molecules.

[B45] Liu B, Liu Y, Yang G, Xu Z, Chen J (2013). Ursolic acid induces neural regeneration after sciatic nerve injury. Neural Regen Res.

[B46] Mlala S, Oyedeji AO, Gondwe M, Oyedeji OO (2019). Ursolic Acid and Its Derivatives as Bioactive Agents. Molecules.

[B47] Ding H, Wang H, Zhu L, Wei W (2017). Ursolic Acid Ameliorates Early Brain Injury After Experimental Traumatic Brain Injury in Mice by Activating the Nrf2 Pathway. Neurochem Res.

[B48] Barbizan R, Castro MV, Rodrigues AC, Barraviera B, Ferreira RS, Oliveira ALR (2013). Motor recovery and synaptic preservation after ventral root avulsion and repair with a fibrin sealant derived from snake venom. PloS One.

[B49] Barros LC, Ferreira RS, Barraviera SRCS, Stolf HO, Thomazini-Santos IA, Mendes-Giannini MJS, Toscano E, Barraviera B (2009). A new fibrin sealant from Crotalus durissus terrificus venom: applications in medicine. J Toxicol Environ Health B Crit Rev.

[B50] Biscola NP, Cartarozzi LP, Ulian-Benitez S, Barbizan R, Castro MV, Spejo AB, Ferreira RS, Barraviera B, Oliveira ALR (2017). Multiple uses of fibrin sealant for nervous system treatment following injury and disease. J Venom Anim Toxins incl Trop Dis.

[B51] Ferreira RS, Barros LC, Abbade LPF, Barraviera SRCS, Silvares MRC, Silvares MRC, Pontes LG, Santos LD, Barraviera B (2017). Heterologous fibrin sealant derived from snake venom: from bench to bedside - an overview. J Venom Anim Toxins incl Trop Dis.

[B52] Barbizan R, Castro MV, Ferreira RS, Barraviera B, Oliveira ALR (2014). Long-term spinal ventral root reimplantation, but not bone marrow mononuclear cell treatment, positively influences ultrastructural synapse recovery and motor axonal regrowth. Int J Mol Sci.

[B53] Irintchev A, Wu MM, Lee HJ, Zhu H, Feng YP, Liu YS, Bernreuther C, Loers G, You SW, Schachner M (2011). Glycomimetic improves recovery after femoral injury in a non-human primate.. J Neurotrauma.

[B54] Abercrombie M, Johnson ML (1946). Quantitative Histology of Wallerian Degeneration.

[B55] Cartarozzi LP, Perez M, Kirchhoff F, Oliveira ALR (2019). Role of MHC-I Expression on Spinal Motoneuron Survival and Glial Reactions Following Ventral Root Crush in Mice. Cells.

[B56] Oliveira ALR, Thams S, Lidman O, Piehl F, Hokfelt T, Karre K, Linda H, Cullheim S (2004). A role for MHC class I molecules in synaptic plasticity and regeneration of neurons after axotomy. Proc Nat Acad Sci U S A.

[B57] Inserra MM, Bloch DA, Terris DJ (1998). Functional indices for sciatic, peroneal, and posterior tibial nerve lesions in the mouse. Microsurgery.

[B58] Carlstedt T, Anand P, Hallin R, Misra PV, Norén G, Seferlis T (2000). Spinal nerve root repair and reimplantation of avulsed ventral roots into the spinal cord after brachial plexus injury. J Neurosurg.

[B59] Barbizan R, Castro MV, Barraviera B, Ferreira RS, Oliveira ALR (2014). Influence of delivery method on neuroprotection by bone marrow mononuclear cell therapy following ventral root reimplantation with fibrin sealant. PloS One.

[B60] Li L, Zhang X, Cui L, Wang L, Liu H, Ji H, Du Y (2013). Ursolic acid promotes the neuroprotection by activating Nrf2 pathway after cerebral ischemia in mice. Brain Res.

[B61] Aldskogius H, Liu L, Svensson M (1999). Glial responses to synaptic damage and plasticity. J Neurosci Res.

[B62] Nimmerjahn A, Kirchhoff F, Helmchen F (2005). Resting microglial cells are highly dynamic surveillants of brain parenchyma in vivo. Science.

[B63] Peruzzotti-Jametti L, Donegá M, Giusto E, Mallucci G, Marchetti B, Pluchino S (2014). The role of the immune system in central nervous system plasticity after acute injury. Neuroscience.

[B64] Araújo MR, Kyrylenko S, Spejo AB, Castro MV, Ferreira RS, Barraviera B, Oliveira ALR (2017). Transgenic human embryonic stem cells overexpressing FGF2 stimulate neuroprotection following spinal cord ventral root avulsion. Exp Neurol.

[B65] Honarvar F, Hojati V, Zare L, Bakhtiari N, Javan M (2022). Ursolic Acid Enhances Myelin Repair in Adult Mice Brains and Stimulates Exhausted Oligodendrocyte Progenitors to Remyelinate. J Mol Neurosci.

[B66] Zhang Y, Li X, Ciric B, Curtis MT, Chen WJ, Rostami A, Zhang GX (2020). A dual effect of ursolic acid to the treatment of multiple sclerosis through both immunomodulation and direct remyelination. Proc Nat Acad Sci U S A.

[B67] Barbizan R, Oliveira ALR (2010). Impact of acute inflammation on spinal motoneuron synaptic plasticity following ventral root avulsion. J Neuroinflammation.

[B68] Sofroniew MV (2015). Astrogliosis. Cold Spring Harb Perspect Biol.

[B69] Sofroniew MV (2005). Reactive astrocytes in neural repair and protection. Neuroscientist.

[B70] Chiarelli N, Zoppi N, Ritelli M, Venturini M, Capitanio D, Gelfi C, Colombi M (2021). Biological insights in the pathogenesis of hypermobile Ehlers-Danlos syndrome from proteome profiling of patients’ dermal myofibroblasts. Biochim Biophys Acta.

[B71] Pedrazzi M, Melloni E, Sparatore B (2010). 3 - Selective Pro-Inflammatory Activation of Astrocytes by High Mobility Group Box 1 Protein Signaling.

[B72] Sroga JM, Jones TB, Kigerl KA, McGaughy VM, Popovich PG (2003). Rats and mice exhibit distinct inflammatory reactions after spinal cord injury. J Comp Neurol.

[B73] Inman D, Guth L, Steward O (2002). Genetic influences on secondary degeneration and wound healing following spinal cord injury in various strains of mice. J Comp Neurol.

[B74] Lima BHM, Bombeiro AL, Cartarozzi LP, Oliveira ALR (2022). The Time Course of MHC-I Expression in C57BL/6J and A/J Mice Correlates with the Degree of Retrograde Gliosis in the Spinal Cord following Sciatic Nerve Crush. Cells.

[B75] Linda H, Shupliakov O, Ornung G, Ottersen OP, Storm-Mathisen J, Risling M, Cullheim S (2000). Ultrastructural evidence for a preferential elimination of glutamate-immunoreactive synaptic terminals from spinal motoneurons after intramedullary axotomy. J Comp Neurol.

[B76] Alvarez FJ, Rotterman TM, Akhter ET, Lane AR, English AW, Cope TC (2020). Synaptic Plasticity on Motoneurons After Axotomy: A Necessary Change in Paradigm. Front Mol Neurosci.

[B77] Blinzinger K, Kreutzberg G (1968). Displacement of synaptic terminals from regenerating motoneurons by microglial cells. Z Zellforsch Mikrosk Anat.

[B78] Rotterman TM, Akhter ET, Lane AR, MacPherson KP, Garcia VV, Tansey MG, Alvarez FJ (2019). Spinal Motor Circuit Synaptic Plasticity after Peripheral Nerve Injury Depends on Microglia Activation and a CCR2 Mechanism. J Neurosci.

[B79] Belov Kirdajova D, Kriska J, Tureckova J, Anderova M (2020). Ischemia-Triggered Glutamate Excitotoxicity From the Perspective of Glial Cells. Front Cell Neurosci.

[B80] Nieke J, Schachner M (1985). Expression of the neural cell adhesion molecules L1 and N-CAM and their common carbohydrate epitope L2/HNK-1 during development and after transection of the mouse sciatic nerve. Differentiation.

